# How low can we go? The implications of low bacterial load in respiratory microbiota studies

**DOI:** 10.1186/s41479-018-0051-8

**Published:** 2018-07-05

**Authors:** Robyn L. Marsh, Maria T. Nelson, Chris E. Pope, Amanda J. Leach, Lucas R. Hoffman, Anne B. Chang, Heidi C. Smith-Vaughan

**Affiliations:** 1 Child Health Division, Menzies School of Health Research, Darwin, Northern Territory Australia; 2 Respiratory Medicine, Seattle Children’s Hospital and University of Washington, Seattle, Washington USA; 3 Department of Respiratory and Sleep Medicine, Children’s Health Queensland and Queensland University of Technology, Brisbane, QLD Australia

**Keywords:** Microbiota, 16S rRNA gene sequencing, Bacterial load, Low bacterial load, Background contamination

## Abstract

**Background:**

Culture-independent sequencing methods are increasingly used to investigate the microbiota associated with human mucosal surfaces, including sites that have low bacterial load in healthy individuals (e.g. the lungs). Standard microbiota methods developed for analysis of high bacterial load specimens (e.g. stool) may require modification when bacterial load is low, as background contamination derived from sterile laboratory reagents and kits can dominate sequence data when few bacteria are present.

**Main body:**

Bacterial load in respiratory specimens may vary depending on the specimen type, specimen volume, the anatomic site sampled and clinical parameters. This review discusses methodological issues inherent to analysis of low bacterial load specimens and recommends strategies for successful respiratory microbiota studies. The range of methods currently used to process DNA from low bacterial load specimens, and the strategies used to identify and exclude background contamination are also discussed.

**Conclusion:**

Microbiota studies that include low bacterial load specimens require additional tests to ensure that background contamination does not bias the results or interpretation. Several methods are currently used to analyse the microbiota in low bacterial load respiratory specimens; however, there is scant literature comparing the effectiveness and biases of different methods. Further research is needed to define optimal methods for analysing the microbiota in low bacterial load specimens.

## Background

The emergence of high throughput sequencing technologies over the past 10 years has enabled deep understanding of the microbiota associated with human mucosal surfaces. Much of this work has focused on the gut, as the digestive tract has the highest microbial density in the human body. As a result, current microbiota methods were largely optimized for analysis of stool specimens [1]. Application of these methods to other specimen types is not necessarily straightforward, as the bacterial load in stool is several orders of magnitude higher than that at other mucosal sites, including the respiratory tract [2, 3]. The potential for large variation in microbial density between different specimen types prompts consideration of how bacterial load may affect microbiota studies. This review considers important implications of low bacterial load in respiratory microbiota studies. The review highlights the importance of residual bacterial DNA as a source of background contamination in sterile laboratory reagents and kits, discusses how this contamination can dominate the microbiota profiles of low bacterial load specimens, and reviews current strategies for overcoming this limitation.

## Why is bacterial load an important consideration when performing microbiota sequencing?

An inverse power relationship exists between the number of bacterial genomes used to prepare sequencing libraries and the proportion of erroneous reads present in microbiota sequence data. This relationship was clearly demonstrated by studies that used microbiota sequencing to analyze pure bacterial cultures. An analysis of serially diluted *Salmonella bongori* culture found that the relative abundance of erroneous reads in the microbiota data progressively increased when < 10^8^ *S. bongori* genome equivalents were sequenced; when ≤ 10^3^ genome equivalents were analysed, > 90% of resulting sequences were not *S. bongori* [4]. Similar findings were reported for pure *Staphylococcus aureus* and *Escherichia coli* cultures [5].

In vitro studies have identified residual 16S rDNA in sterile laboratory reagents and DNA extraction kits as the primary source of background contamination in microbiota data [4–6]. Taxa suggestive of environmental contamination have also been detected in respiratory specimens with low bacterial load, but not in the corresponding negative controls [7, 8]. In one study, environmental taxa with relative abundance inversely proportional to total bacterial load (strongly suggestive of background contamination) were detected exclusively in the clinical specimens [7]. It is currently unclear whether detection of such taxa indicates unsampled sources of background contamination or unidentified amplification and sequencing errors [9, 10].

To date, more than 200 genera have been reported as background contamination in microbiota data from negative controls and low bacterial load specimens (Table) [4, 8, 11]. This list includes genera previously reported amongst the microbiota of low bacterial load human mucosal and tissue specimens [12, 13]. The potential for misinterpretation of background contamination in data from low bacterial load specimens was recently highlighted in placental microbiota studies [14]. Early reports suggested the presence of a commensal placental microbiota with potential impacts for the developing infant microbiome [15, 16]; however, these early studies were limited, as tests for background contamination were not performed. This limitation was emphasized by a subsequent study that included robust negative controls and bacterial load measures and found that the placental microbiota of women with uncomplicated pregnancies could not be differentiated from background contamination present in negative controls [17]. This example highlights the importance of bacterial load measures and negative controls when studying the microbiota of environments or specimens with potentially low numbers of bacteria. Other examples of anatomic sites that are expected to have low bacterial load or be sterile in the absence of clinical infection include the middle ear [18], eyes [19], urinary system [13], central nervous system [20] and the lower airways [3].

## Background contamination in respiratory microbiota studies

Several studies have reported background contamination in microbiota data generated from respiratory specimens with low bacterial load (< 10^6^ bacterial cells/mL of specimen) [3, 4, 7, 8, 18, 21]. Factors that determine the bacterial load in respiratory specimens include the specimen type (e.g. sputum, bronchoalveolar lavage [BAL], swabs), the specimen volume, the anatomic site sampled (e.g. upper or lower airways) and clinical parameters (e.g. age, acute infection, exacerbation, antibiotic use).

Many respiratory microbiota studies have analyzed sputum from patients with lower airway disease. Spontaneously expectorated sputum has relatively high bacterial load (typically > 10^8^ 16S copies per gram or mL sputum [22–24]), and thus, sputum microbiota data are expected to be less affected by background contamination than specimens containing lower bacterial densities (e.g. BAL specimens).

The bacterial load reported for BAL specimens varies depending on the volume tested and the disease state. BAL from healthy adults has low bacterial load (< 10^6^ 16S copies/mL) [3, 25], and thus, concentration of cellular components from large BAL volumes (> 100 mL) may be needed prior to DNA extraction to avoid background contamination issues [26]. When lower BAL volumes (< 2 mL) [3] or acellular BAL [25] were tested, taxa consistent with background contamination were present in the microbiota data. BAL bacterial load may also vary in patients with lower airways disease. For example, high BAL bacterial load (up to 10^8^ 16S copies/mL) was reported for patients with cystic fibrosis [27] and interstitial pulmonary fibrosis [28], whereas low bacterial load was reported for BAL from children with protracted bacterial bronchitis or bronchiectasis [7].

Studies of oropharyngeal swabs have reported bacterial load at levels high enough to minimise adverse effects from background contaminants (> 10^6^ 16S copies/mL) [3, 7, 27]; however, lower bacterial load (and therefore higher risk of bias due to detection of background contamination) was reported following antibiotic treatment [27]. Nasopharyngeal (NP) bacterial load is lower than that in the oropharynx, and varies depending upon the age [29] and disease status [30] of the study population. Several microbiota studies have reported low bacterial load in a proportion of NP specimens [18, 31, 32], with some studies reporting low bacterial load in up to a third of the NP swabs tested [7, 21].

## What can be done to reduce background contamination effects in respiratory microbiota studies?

Given the potential for variation in the amount of bacteria present in clinical specimens, this review recommends that quantitative polymerase chain reaction (qPCR)-based measures of bacterial load [33] be included in respiratory microbiota studies: firstly, to aid investigation of background contamination issues [17]; and secondly, to allow critical review of published data. Here, we discuss key stages in the design and implementation of microbiota analyses that can be affected by bacterial load issues.

### Study design and specimen collection

Establishing a standardized methodology for low bacterial load specimens requires consideration of differences in patient populations and specimen types. Many microbiota studies are designed to compare the microbiota present at high bacterial load in all study groups, e.g. gut microbiota studies. In respiratory studies, bacterial load can be more variable with low bacterial load specimens more common in some groups than others. For example, lower airway microbiota in healthy controls compared to that of patients with chronic lung disease [34], or bacterial load variations between groups defined by antibiotic exposure [27, 35], or respiratory sites [7, 31]. Bacterial load is also important because specimens containing few bacteria may be excluded from downstream analyses if insufficient amplicons or sequence reads are generated. This exclusion risks loss of statistical power by reducing the sample size or by introducing imbalances to the study design that may affect downstream analyses.

Optimal specimen type and volume should also be considered when planning respiratory microbiota studies. For example, in lower airway studies where sputum collection is not possible, protected bacterial brush specimens may yield higher bacterial load than BAL [3], and thus may help abrogate background contamination effects. In BAL studies, recovery of bacterial DNA can be improved by concentrating large sample volumes [26]; however, this may not be possible where lower saline volumes are instilled (e.g. paediatric studies [36]) or when limited BAL volume is available. Opportunities to increase the volume of upper airway specimens can be limited, especially when swabs are collected. Swab-based respiratory microbiota analyses are typically performed using < 0.5 mL of the preservative media [7, 21, 29, 37]. Increasing this sample volume (e.g. by using the entire swab [31]) will improve recovery of bacterial DNA; however, it is important to note that linear increments in the swab volume will not achieve the log_10_-scale increases in bacterial load that may be required to minimize the proportion of background contamination in microbiota data.

### Quality control

Where low bacterial load specimens are unavoidable, care must be taken to avoid introduction of exogenous DNA during specimen collection and laboratory processing. This includes taking steps to avoid introduction of exogenous DNA originating from the airways of laboratory staff (e.g. processing of specimens in biosafety cabinets). Negative controls should be processed alongside clinical specimens through all analytic stages (including specimen collection and sequencing) to ensure all potential sources of background contamination are sampled [1, 4, 17, 38]. Relevant negative controls include those that sample any equipment or media used during specimen collection. For BAL studies, bronchoscope saline wash controls should be collected prior to insertion into the airways, as these controls can be critical to the appropriate interpretation of microbiota data when bacterial load is low [3, 31] or when it overlaps with that of negative controls [25]. Negative controls should be included through all stages of laboratory processing, including sequencing [4, 17]. Where possible, nucleic acid-free laboratory reagents should be used. It is also recommended that laboratory processing be done in clean environments (e.g. decontaminated and UV-treated hoods) using dedicated reagents and pipettes to minimise contamination [1, 4]. Minimizing the number of liquid handling events during DNA extraction may help to reduce introduction of exogenous DNA and may also improve yield by preventing DNA loss during processing. Given the potential risks for confounding of microbiota data by background contamination, there is growing recognition of the need for negative control results and bacterial load measures to be included when publishing microbiota data from low bacterial load specimens [17, 38].

### Optimizing DNA extraction for low bacterial load specimens

A range of DNA extraction methods have been used to prepare respiratory specimens for microbiota analysis; however, few studies have compared the bacterial DNA yield achieved by different methods. Biesbroek et al. [21] reported a 100-fold difference in bacterial DNA yield between extraction methods when applied to both high and low bacterial load upper airway specimens. This study reported a magnetic bead-based DNA purification method outperformed commonly used silica columns, including the MoBio Powersoil DNA Isolation kit (Qiagen Pty Ltd., Dandenong, Australia), which is widely used and has been recommended by large international sequencing projects (e.g. the United States National Institutes of Health Human Microbiome Project [39]).

Optimal DNA extraction pre-treatment conditions are not well defined for low bacterial load specimens. Mechanical disruption by bead-beating is widely accepted to improve bacterial coverage by lysing cells that resist chemical or enzymatic processes [21, 40]; however, some studies have reported reduced DNA yield following mechanical disruption [41, 42], potentially due to DNA shearing. Bead-beating has been combined with chemical and/or enzymatic lysis; however, optimal pre-treatment conditions have not been determined or standardized. Some studies have included bead-beating with phenol-chloroform extraction methods [21, 43]; however, these methods can be difficult to perform (risking loss of specimen volume), include toxic chemicals and are not easily automated. Other studies have combined bead-beating and enzymatic digestion to improve bacterial coverage [29, 44]; however, there is little standardization in either the types of enzymes used or whether the enzymatic digestion is performed before or after bead-beating. Further research is needed to inform optimal pre-treatment strategies for maximum recovery and coverage of bacterial DNA from low load respiratory specimens.

Variation in background contamination among batches of DNA extraction kits or reagents must also be considered when studying the microbiota in low bacterial load specimens. Salter et al. [4] reported batch-specific variation in background contamination from DNA extraction kits used to longitudinally assess NP microbiota in children. In this study, NP swabs were processed in chronological order, with different batches of the DNA extraction kit used for specimens from different time-points. Initial analyses suggested age-specific changes in the NP microbiota; however, these associations were not detected once the batch-specific background contamination was removed [4]. To prevent this type of confounding, it is recommended that a single batch of DNA extraction reagents is used when analyzing the microbiota of low bacterial load specimens [4]. Where use of multiple batches of extraction reagents cannot be avoided, it is recommended that specimens are processed in a random order with concurrent processing of batch-specific negative controls [4].

### Optimizing 16S rDNA amplification from low bacterial load specimens

Even with optimized, high-performing DNA extraction methods, there may be instances where bacterial load is low. For example, diminished loads may result from testing low volume specimens from healthy lower airways. In these instances, optimization of the 16S rDNA PCR may be needed to achieve sufficient amplicons for sequencing.

Many different strategies have been used to increase amplicon yield from low bacterial load respiratory specimens. Some studies have improved the amplicon yield by using > 30 PCR cycles [21]; however, this approach is not recommended as it can increase amplification errors [45, 46] as well as increasing detection of background contamination [4]. Some respiratory studies [31, 47] have used touchdown-PCR in which the annealing temperature is varied to improve amplicon specificity [48]; however, this method may not improve amplicon yield. Several nested PCRs have also been used in studies where a single round of PCR did not generate sufficient amplicons [49–52]. Nested PCR is expected to improve amplicon yield and also to reduce heteroduplex and chimera formation [9], but requires additional liquid handling steps which risks further introduction of exogenous DNA. Whole specimen amplification techniques (e.g. multiple displacement amplification) may also be used to increase the amount of DNA available for sequencing studies [53, 54]; however, this method may increase the overall sequencing error rate.

As with DNA extraction methods, there is currently scant literature comparing the strengths and weaknesses of different approaches for increasing amplicon yield, including assessment of the impacts of different amplification strategies on overall sequence error rates. It is also important to recognize that while optimized amplification methods may improve amplicon yield, background contamination issues are likely to remain as exogenous DNA present in sampling equipment and laboratory reagents will be co-amplified alongside the target DNA.

### Optimizing library preparation

16S sequencing libraries are routinely prepared using a standardized input mass of PCR amplicons. The Earth Microbiome Project (www.earthmicrobiome.org) currently recommends library preparation with 240 ng of amplicons from each sample [55]; however, this amount may be difficult to generate when applying standard protocols to respiratory specimens, especially when the bacterial load is low [4]. To overcome this limitation, some studies have excluded specimens generating too few amplicons when preparing sequencing libraries [56, 57]. Others have successfully used a lower mass of amplicons to prepare the library (25–100 ng) [5, 58]. A third approach has been to pool multiple PCR replicates of low-load specimens to ensure a minimum amplicon mass is achieved. This approach was successfully used by Salter et al. [8] to characterize the microbiota in NP swabs from infants; however, up to 18 replicates were needed for some specimens.

### Identifying and excluding background contamination

As discussed above, microbiota studies that include specimens with low bacterial load need to test for and exclude background contamination prior to data analysis. Failure to identify and exclude background contamination risks confounding of the microbiota analysis and can result in incorrect conclusions, as recently demonstrated by studies of the placental [14, 17] and nasopharyngeal [4] microbiota.

The first step in assessing background contamination is to ensure that negative controls are included through all stages of specimen collection and laboratory processing [4, 17, 38]. Although negative controls and low bacterial load specimens may generate low amplicon yields, it is recommended that these specimens not be excluded from sequencing libraries to ensure that background contamination is fully sampled [17, 38]. It is also recommended that microbiota studies report results of negative controls and bacterial load data, especially when specimens with low bacterial load are tested [17, 38].

The second step is to review sequence data from negative controls and clinical specimens to identify and exclude background contamination. Several studies have removed background contamination by excluding all operational taxonomic units (OTUs) identified in negative controls [47, 52, 59, 60]; however, this approach risks exclusion of biologically significant OTUs that may be present in negative controls as a result of barcoding errors [5, 8, 11, 61, 62]. For example, a *Haemophilus* read from a clinical specimen may have a barcode error that results in it being incorrectly assigned to a negative control; exclusion of all OTUs detected in negative controls would exclude this taxon from downstream analyses. This is an important point, as OTUs consistent with constituents of the human respiratory microbiota have been detected in negative controls (e.g. *Streptococcus, Haemophilus, Pseudomonas* and *Burkholderia*, Table 1). Thus, summary exclusion of all OTUs detected in negative controls may impact the accuracy of microbiota data from the clinical specimens.

**Table 1:** Examples of genera detected previously as background contamination in negative controls from respiratory microbiota studies [4, 8, 11, 52]

Abiotrophia^a^	Bifidobacterium	Curvibacter^a^	Halocella	Microlunatus	Pseudoclavibacter	Staphylococcus
Acidocella	Blastococcus	Deinococcus	Herbaspirillum^a^	Moryella	Pseudoflavonifractor	Stenotrophomonas^a^
Acidovorax^a^	Blautia	Delftia	Hoeflea	Neisseria	Pseudomonas^a^	Streptococcus^a^
Acinetobacter^a^	Bosea	Devosia	Hydrotalea^a^	Nevskia	Pseudorhodoferax	Sulfuritalea^a^
Actionmyces	Bradyrhizobium^a^	Dialister	Ilumatobacter	Niastella	Pseudoxanthomonas^a^	Suttonella
Adhaeribacter	Brevibacillus^a^	Dietzia^a^	Intestinibacter	Nocardioides	Psychrobacter	Tericibacter
Aerococcus	Brevibacterium	Dorea	Janibacter	Novosphingobium	Quinella	Thauera
Aeromicrobium^a^	Brevundimonas^a^	Duganella	Janthinobacterium^a^	Ochrobactrum^a^	Ralstonia^a^	tm7
Afipia^a^	Brochothrix	Dyadobacter	Johnsonella	Olivibacter^a^	Rhizobium^a^	Treponema
Agrococcus	Brooklawnia	Dyella	Kingella^a^	Oscillospira	Rhodococcus^a^	Trichococcus
Alcaligenes	Burkholderia^a^	Eggerthella	Klebsiella	Oxalobacter^a^	Roseateles	Tsukamurella
Alcanivorax	Cadidatus pelagibacter	Elusimicrobium	Kocuria	Paenibacillus^a^	Roseburia	Tumebacillus
Alicycliphilus	Cadidatus_alysiospaera	Enhydrobacter^a^	Lachnoanaerobaculum	Papilibacter	Roseomonas^a^	Tyzzerella
Alistipes	Campylobacter	Enterobacter	Lactobacillus	Parabacteroides	Rothia	Unclassified Gp 2 Acidobacteria
Aminobacter	Capnocytophagia	Enterococcus	Lactococcus	Paracoccus^a^	Rubellimicrobium	Undibacterium^a^
Anaerococcus	Cardiobacterium^a^	Erysipelatoclostridium	Lanoclostidium	Parasutterella	Ruegeria	Vampirovibrio
Anaeromyxobacter	Catenibacterium	Escherichia_Shigella^a^	Lanospira	Parvimonas	Ruminococcus	Variovorax^a^
Anaerotrucus	Caulobacter^a^	Eubacterium	Leptothrix	Patulibacter	Runiniclostridium	Veillonella^a^
Anoxybacillus	Cellvibrio	Facklamia	Leptotrichia	Pedobacter^a^	Saccharopolyspora	Wautersiella
Aquabacterium^a^	Christensenella	Faecalibacterium	Limnobacter	Pedomicrobium	Salinicoccus	Xanthomonas
Arthrobacter^a^	Chryseobacterium^a^	Fastidiosipila	Massilia^a^	Pelomonas^a^	Schlegelella	Xlyanibacter
Asticcacaulis	Cloacibacterium	Flavobacterium	Megasphaera^a^	Peptococcus	Seleomonas	
Atopobium	Clostridium	Flavonifractor	Meiothermus	Petoniphilus	Serratia	
Aurantimonas^a^	Collinsella	Fusobacterium	Mesorhizobium^a^	Phyllobacterium^a^	Shewanella	
Azoarcus	Comamonas^a^	Gemella	Methylobacterium^a^	Pilimelia	Slackia	
Azospira	Coprococcus	Geobacillus	Methylophilus	Polaromonas	Solobacterium	
Bacillus^a^	Corynebacterium^a^	Geodermatophilus	Methylopila	Porphyromonas	Sphingobacterium^a^	
Bacteroides	Craurococcus	Gordonia	Methyloversatilis	Prevotella^a^	Sphingobium^a^	
Beijerinckia	Cupriavidus	Granulicatella^a^	Microbacterium	Propionibacterium^a^	Sphingomonas^a^	
Beutenbergia	Curtobacterium	Haemophilus	Micrococcus	Pseudobutyrivibrio	Sphinogpyxis	

It is important that negative controls are included in all studies of the microbiota in low bacterial load specimens, as background contamination in sterile reagents and kits is likely to be batch-specific

^a^Indicates genera detected as contaminants in multiple studies

Many different strategies have been used to overcome this limitation, each of which has different strengths and weaknesses (Table 2). Salter et al. [8] identified background contaminants by conservatively assessing OTUs detected in negative controls to avoid exclusion of biologically relevant taxa, as well as reviewing data from replicate DNA extractions and different DNA extraction batches. Lazarevic et al. [63] sequenced serial dilutions of pure bacterial cultures and bioinformatically tested for background contamination by determining the ratio of mean OTU abundance in negative controls compared to data from the culture specimens, then excluding OTUs with ratios > 0.001 as probable contaminants. Jervis-Bardy et al. [56] excluded OTUs detected in negative controls or clinical specimens when a strong negative correlation was observed between the relative abundance and the bacterial load. Bosch et al. [32] used complete linkage hierarchical clustering to identify and exclude clinical specimens with microbiota profiles indistinguishable from negative controls. Venkataraman et al. [64] used a neutral model to identify probable background contamination reads in microbiota data from respiratory specimens with low bacterial load; reads that were identified by the neutral model as likely to have originated from background contamination were excluded prior to OTU clustering.

**Table 2:** Summary of methods used to identify and exclude background contamination in microbiota datasets

Method	How does it work?	Strengths and weaknesses
Exclusion method (e.g. [47, 52])	All OTUs detected in negative controls are excluded as background contaminants	Background contaminants will be excluded; however, the method is not recommended as it risks exclusion of biologically relevant OTUs that can occur in negative controls (e.g. because of barcoding errors) [5, 8, 11, 61, 62].
Replicate method [8]	Contaminant OTUs excluded based on replicate data from different DNA extraction batches and negative controls.	This method is not suitable if replicate extractions are not possible. Requires subjective interpretation of negative control data.
Abundance ratio [5]	The ratio of mean OTU abundance in negative controls and study specimens is calculated. OTUs with a ratio > 0.001 are excluded as probable background contaminants.	Unsupervised method developed using pure bacterial cultures. Optimal threshold for defining contamination in more complex microbiota data may need to be determined.
Correlation analysis [56]	Spearman correlation between OTU relative abundance and bacterial load is determined. OTUs showing a strong and significant negative correlation with bacterial load (Spearman rho < − 0.7) are excluded as probable contaminants.	Unsupervised method. Limited when applied to OTUs present in < 20 specimens. Significance testing requires adjustment for multiple measures.
Cluster analysis [32]	Hierarchical cluster analysis is used to identify and exclude specimens with high similarity to negative controls.	Unsupervised method. Exclusion of specimens (instead of reads or OTUs) may impact study design. Background contamination clusters may be difficult to define where similarity between negative controls and low bacterial load specimens is variable.
Neutral model [64]	Reads that are neutrally distributed and enriched in negative controls are excluded prior to OTU clustering. Additionally, only reads that are unique to clinical specimens are included in downstream analyses.	Unsupervised method. It is unclear whether restriction of downstream analyses to reads unique to clinical specimens may result in exclusion of biologically relevant taxa (e.g. because of barcoding errors).

The authors are unaware of any studies to date that have compared the strengths and weaknesses of these different methods. In the absence of such comparisons, the authors currently recommend using one of the unsupervised methods that exclude contaminant reads or OTUs (Table 2). Cluster analysis may be appropriate; however, this method requires careful consideration of the impact of specimen exclusion on the study design and may be problematic if negative control data do not form a distinct cluster. Inclusion of mock community controls at different bacterial loads to enable assessment of the selected contaminant exclusion strategy is recommended. It is not recommended to exclude all OTUs detected in negative controls, as this risks exclusion of potentially biologically relevant OTUs present in negative control data because of sequencing errors (e.g. barcoding errors).

## Current recommendations

Salter et al. [4] and Lauder et al. [17] systematically assessed background contamination in microbiota data from low bacterial load specimens and made a series of recommendations that help define current best practice. In combination, these recommendations are as follows:

1. Negative controls should be processed alongside clinical specimens through all stages of laboratory testing (including specimen collection and sequencing) to ensure that all potential sources of exogenous DNA are sampled.
2. Where high volume specimens are collected (e.g. BAL from adult patients), the specimen should be concentrated prior to DNA extraction to maximise recovery of bacterial DNA.
3. Use of a single batch of DNA extraction reagents. If this is not possible, specimens should be processed in a random order to reduce the risk of false pattern formation, with negative controls processed with each batch.
4. Where possible, include replicate DNA extractions and sequencing.
5. Bacterial load should be determined prior to sequencing to determine whether specimens with low bacterial load are present.
6. Negative controls should be sequenced to maximise sampling of background contamination.
7. Testing to identify and exclude taxa suggestive of background contamination should be done prior to downstream analyses of microbiota from clinical specimens.

This review recommends that these parameters are reported in a standardized manner when publishing microbiota data. It is also recommended that the following parameters be detailed when publishing data from specimens with potentially low bacterial load:

1. The types of negative controls tested.
2. The methods used to collect and process negative controls.
3. Negative control results and bacterial load data.
4. The methods used to identify and exclude background contamination.
5. A list of taxa and/or OTUs excluded prior to downstream analyses.
6. Results of any downstream testing used to confirm the presence of biologically unexpected taxa (e.g. extended culture, species-specific PCR, fluorescent in situ hybridization [FISH]).

## Summary

Modern DNA sequencing technologies provide previously unimagined capacity to interrogate the complex bacteriology present on human mucosal surfaces; however, it is increasingly recognized that standard methods require modification when applied to low bacterial load specimens. As bacterial load in respiratory specimens can be highly variable, it is critical that respiratory microbiota studies include robust negative controls and report bacterial load measures. Where low bacterial load specimens are analyzed, it is essential that background contamination is tested and accounted for and, if possible, excluded to avoid confounding. A range of methods are currently used to process DNA from low bacterial load specimens and to identify and exclude background contamination; however, there is scant literature comparing the effectiveness and biases of different methods. Priority areas for future research include studies to determine optimal methods for analysis of low bacterial load specimens, including specimen collection parameters, DNA extraction and PCR amplification conditions, and assessment of the different bioinformatic methods used to identify and exclude background contamination.

## Abbreviations

BAL: Bronchoalveolar lavage
FISH: Fluorescent in situ hybridization
OTUs: Operational taxonomic units
PCR: Polymerase chain reaction
qPCR: quantitative PCR
